# Trace element levels in the muscles of three tern species (Aves: Laridae) from the western Arabian Gulf: environmental assessment and implications for conservation

**DOI:** 10.1007/s10661-024-12385-9

**Published:** 2024-02-05

**Authors:** Lamia Yacoubi, Dario Savoca, Radhouan Belgacem El Zrelli, Jinoy Gopalan, Mazen Nazal, Yu-Jia Lin, Antonella Maccotta, Foued Hamza, Md. Simul Bhuyan, Marco Arculeo, Lotfi Jilani Rabaoui

**Affiliations:** 1 Faculty of Science of Tunis, Laboratory of Biodiversity & Parasitology of Aquatic Ecosystems (LR18ES05), University Campus, University of Tunis El Manar, 2092 Tunis, Tunisia; 2 Dipartimento di Scienze e Tecnologie BiologicheChimiche e Farmaceutiche (STEBICEF), Università degli Studi di Palermo, Via Archirafi 20, 90123 Palermo, Italy; 3 NBFC, National Biodiversity Future Center, 90133 Palermo, Italy; 4 SADEF Agronomy & Environment, 30 Rue de la Station, 68700 Aspach-Le-Bas, France; 5 Applied Research Center for Environment and Marine Studies, Research Institute, King Fahd University of Petroleum and Minerals (KFUPM), 31261 Dhahran, Saudi Arabia; 6 Institute of Oceanography, National Taiwan University, Taipei, 10617 Taiwan; 7 National Center for Wildlife, Ministry of Environment, Water & Agriculture, Riyadh, Saudi Arabia; 8 Bangladesh Oceanographic Research Institute, Cox’s Bazar, 4730 Bangladesh

**Keywords:** Trace elements, Marine pollution, Migratory seabirds, Contamination, Conservation, Arabian/Persian-Gulf

## Abstract

In the Arabian Gulf (called also Persian Gulf; hereafter 'the Gulf'), Jana and Karan Islands are recognized as one of the most Important Bird Areas in the region. Many migratory breeding seabirds, like the Greater Crested Tern *Thalasseus bergii*, White-cheeked Tern *Sterna repressa* and Bridled Tern *Onychoprion anaethetus*, depend on these islands during the breeding season. However, these aquatic wildlife species are suffering from intensified urban and industrial coastal development and various contamination events including wars and related oil spills. In this study, we used these three piscivorous top predator birds to analyse the levels of 19 trace elements (TEs; i.e. Al, As, Ba, Ca, Cd, Co, Cr, Cu, Fe, Hg, K, Mg, Mn, Na, Ni, Pb, Sr, V and Zn) in 15 muscular tissue samples from Jana and Karan Islands. PERMANOVA analysis showed no difference in contamination profile between sites nor between species probably due to their spatial and ecological proximity and therefore similar levels of exposure to TEs. Comparing these levels with existing literature, our results showed no particular concern for all elements, except for Al (maximum values recorded = 116.5 µg g^−1^ d.w.) and, in two samples, Ba (33.67 µg g^−1^ d.w.) and Pb (5.6 µg g^−1^ d.w.). The results can be considered as an initial step for supplementary evaluations with a larger number of samples and specified time intervals for the collection of specimens. This study provided baseline information on the pollution status of these two ecologically important sites which require a continuous biomonitoring programme.

**Supplementary information:**

The online version contains supplementary material available at 10.1007/s10661-024-12385-9.

## Introduction

Trace elements (TEs) pollution is an environmental alteration of natural and anthropic origin that affects ecological systems (Bat et al., 2022; Bhuyan et al., 2019). Contaminant emissions have increased due to the intensification and diversification of anthropogenic pressures leading to wider and more widespread exposure and thus to a greater likelihood of contamination resulting in a major change in marine and coastal balance ecosystems (Ali et al., 2022; Kubra et al., 2022). The bioaccumulation of TEs can lead the exposed organisms to negative effects including those of toxicological and genotoxic relevance, from the carcinogenic potential to reproductive dysfunction and behavioural changes even to death (Briffa et al., 2020; Michelutti et al., 2010). These effects depend on the degree of contamination and the species of organisms exposed to TEs types, some of which may be highly toxic even at low concentrations. The toxicity of some elements (e.g. metals or semimetal) is influenced by some factors, for example, their valence status, their combination with organic molecules, the presence of metallothionein in organisms and the environmental pH (Briffa et al., 2020; Ding et al., 2022). Many studies reported TEs contamination in several organisms and habitats, especially in marine ecosystems (Savoca et al., 2022).

Aquatic avifaunal communities are particularly exposed to various pollutants and, due to ecological habits, can accumulate high levels of TEs in their tissues and eggs (Khademi et al., 2015). In addition, negative effects on offspring were observed in embryonic development and hatching success, linked to the presence of TEs such as Cr, Pb and Cd in avian species such as mallard (Thongcharoen et al., 2018). Moreover, seabirds, which accumulate TEs in nesting and feeding sites, may act as bio-vectors of TEs whose efficacy varies with trophic position (Michelutti et al., 2010).

Among seabirds, terns have been the subject of different environmental studies showing that *Laridae* are excellent bioindicators of TEs pollution (Khademi et al., 2015; Kitowski et al., 2018; Korbecki et al., 2019; Michelutti et al., 2010; Zamani-Ahmadmahmoodi et al., 2014). However, these latter studies did not take into consideration all types of TEs nor all tern species. Furthermore, some geographical sites tend to be more contaminated than others because of their characteristics or anthropogenic events. It is, for example, the case of the Arabian Gulf (called also the Persian Gulf, hereafter “the Gulf”) which has known since the 1980s increasing coastal development and various contamination events including wars (e.g. Gulf war of 1991 and 2003) and related oil spills (Khademi et al., 2015; Rabaoui et al., 2020). In addition, the presence of various industrial and residential areas and the semi-enclosed nature of the Gulf contribute to its continuous contamination and the persistence of pollutants accumulating in it (Basyoni, 1999; Khademi et al., 2015; Maneja et al., 2021; Rabaoui et al., 2020).

In the Gulf, Jana and Karan Islands (Is.) are among the most important feeding and nesting areas for many marine wildlife species (Maneja et al., 2021; BirdLife International, 2023). These Is. support important breeding populations of sea turtles, namely, *Eretmochelys imbricata* and *Chelonia mydas*. Jana and Karan Is. are among the most important sites for seabirds (BirdLife International, 2023). These islands play an important role for several seabird species during the breeding season. They support a large number of breeding pairs of species of international importance, such as the Greater Crested Tern (*Thalasseus bergii*), White-cheeked Tern (*Sterna repressa*) and Bridled Tern (*Onychoprion anaethetus*; BirdLife International, 2023). These latter species are recognized as migratory breeding birds in the western part of the Gulf.

Tern species are considered ideal bioindicators because they are long-lived and numerous, live on the coast and on marine habitats are high-level predators, and feed on potentially contaminating preys (Khademi et al., 2015). Jana and Karan Is. are also used as a stopover site by considerable numbers of migratory birds, especially during spring migration (BirdLife International, 2023). Since 2013, Jana and Karan Is. being are part of an Important Bird and Biodiversity Area called the Gulf coral islands (BirdLife International, 2023). Despite the importance of these Is., almost nothing is known about the environmental situation in these important biodiversity areas. Thus, it is important to study some bioindicators to understand the current status of pollution (Khademi et al., 2015).

In the present work, a screening of biomonitoring of 19 TEs in the muscular tissues of the three species of tern (*T. bergii, S. repressa* and *O. anaethetus*), from Jana and Karan Is. in Saudi Arabia has been conducted. The elements analysed were arsenic (As), barium (Ba), calcium (Ca), cadmium (Cd), cobalt (Co), chromium (Cr), copper (Cu), iron (Fe), mercury (Hg), potassium (K), magnesium (Mg), manganese (Mn), sodium (Na), nickel (Ni), lead (Pb), strontium (Sr), vanadium (V), and zinc (Zn).

The primary objectives of this study are to evaluate, for the first time, the concentrations of selected TEs in the tern species residing on Jana and Karan Is., compare these levels with existing literature and assess the potential environmental implications. By addressing these objectives, this study contributes to a deeper understanding of TEs pollution in Gulf, shedding light on the potential risks to avian populations and their habitats.

## Materials and methods

### Study area and sampling

**Fig. 1:**
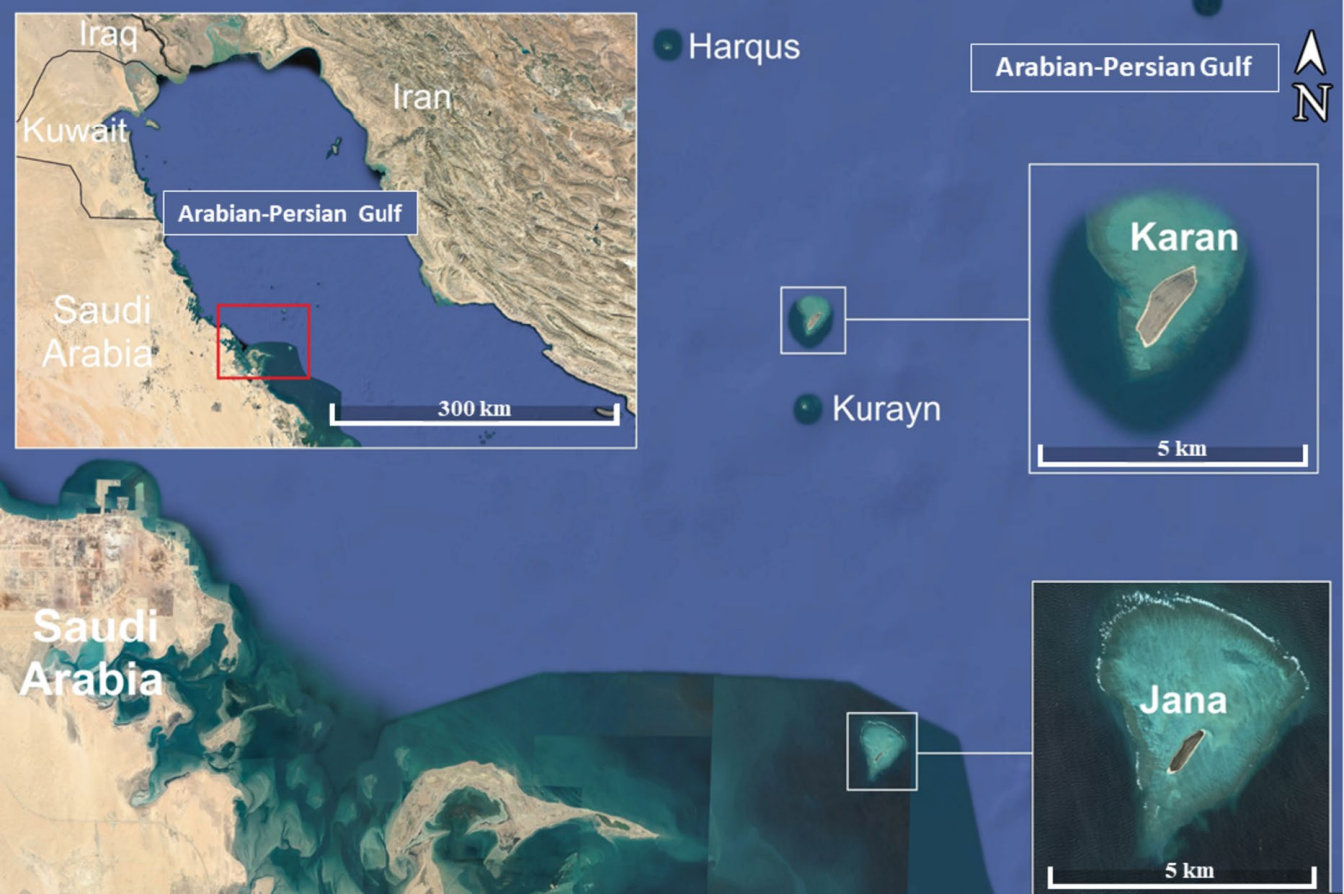
Map showing the location of the Jana and Karan Islands from where the freshly dead tern specimens were sampled

During a scientific prospection conducted, in January 2020, in the offshore Jana (27°22′6.85″N, 49°53′50.85″E) and Karan (27°43′5.05″N, 49°49′28.91″E) Is. (Saudi waters of the Gulf; Fig. 1), muscle tissue samples were taken from freshly dead specimens of *T. bergii*, *S. repressa*, and *O. anaethetus*.

The two coral reef Islands of Jana and Karan cover surface areas of 261 and 970 km^2^, respectively (Maneja et al., 2021), are of naturalistic interest and are known to host many seabird species, especially terns. A team of scientists camped for five days on each of these two islands and ensured to collection daily freshly dead terns. To make sure that only freshly dead animals were collected, the first camping day was dedicated to prospect the coastline and cleaning it of all previously dead terns, which were not taken into consideration in this work. The team started collecting tern samples on the second camping day and continued to do so up to the last day of the camping period. In total, fifteen muscle tissue samples (of 50–70 g each) were collected from the pectoral body zone of freshly dead specimens of *T. bergii* (seven samples), *S. repressa* (three samples) and *O. anaethetus* (five samples). While the three tern species were sampled from Jana Is., only *T. bergii* and *O. anaethetus* were sampled from Karan Island. All tern specimens were adults and were sampled regardless of their gender. The collected muscle tissue samples were separately preserved in glass jars and immediately transported to the laboratory to proceed with the elemental analyses.

### Sample preparation and analysis of elements in biota tissue

Approximately 0.5 g of freeze-dried tissue samples were weighed and transferred into sample digestion vials. For each sample, 10 mL of distilled concentrated nitric acid (HNO_3_) and 2 mL of concentrated perchloric acid (HClO_4_) were added. Then, the samples were kept overnight at room temperature. Later, the samples were heated at 95 °C on a hot block to near dryness. Successively 5 mL of concentrated HNO_3_ were added, and the mixture was heated again until the fumes disappeared. The samples were allowed to cool, and the volume was made up to 25 mL using 2% HNO_3_ in clear PP/PE tubes. The resulting solutions were filtered through Whatman 42 filter paper and analysed following US EPA method 6010 (U.S. EPA Method 6010D -2014) for the sake of quantification of 18 elements (i.e. Al, As, Ba, Ca, Cd, Co, Cr, Cu, Fe, K, Mg, Mn, Na, Ni, Pb, Sr, V and Zn) using an Inductively Couple Plasma-Optical Emission Spectrometry (ICP-OES) instrument (Perkin Elmer DV8000, USA), while Hg was analysed in the bird tissue samples using a direct Hg analyser Nippon 3000 MA. Briefly, 200 mg of the sample was directly burned in an oxygen-rich atmosphere, and thus released Hg was trapped in gold particles. The amalgamated Hg was then heated to release Hg vapour to be measured with an Atomic Absorption Spectrometer following U.S. EPA Method 7473.

### Quality control procedure

For the collection of the specimens, a standardized protocol was applied to ensure that fresh specimens were collected, stored, and preserved in the same condition. For the process of the raw samples, a standardized protocol was applied to avoid contamination from the process, such as the use of a ceramic knife for skin removal and tissue collection and the use of a glass platform and glass container (previously decontaminated) for processed muscular tissue samples.

The integrity of the chemical analysis was ensured by implementing quality control and quality assurance checks as per the USEPA protocols. Laboratory reagent blanks and laboratory calibration standards were run routinely. After every ten samples, the continuous calibration standard was used to check the calibration of the instrument. For every batch of 15 samples, two duplicate samples, one Matrix Spike sample and one Matrix Spike Duplicate, were also run. The spiking of the samples was in the range of 1–5 times the analyte concentration. The percentage recovery was calculated based on spiking, and the limit of recovery was set in the range of 70–130%, as per the USEPA recommendation (U.S. EPA, 2018), for the acceptance of the test results.

The analytical accuracy and precision of trace element analyses (specifically for Al, As, Cd, Co, Cr, Cu, Fe, Hg, Mn, Ni, Pb, Zn) were evaluated using the DORM-2 certified standard reference materials (Dogfish muscle) provided by the Canadian National Research Council (NRC). Most of the ratios are within the U.S. EPA recommendations, which is 100 ± 30%, except for As, which was 138% (Table S1 in Supplementary Information).

### Statistical analyses

A permutational multivariate analysis of variance PERMANOVA was performed to evaluate the significance of differences in the contamination profile between sites or between species. The experimental design comprised two factors [site (two levels, fixed and orthogonal) and species (three levels, fixed and orthogonal)] and 14 variables corresponding to the elements detected. Each term in the analysis was tested by 999 random permutations. For statistical analysis, when no elemental concentration values were detected (i.e. below the limit of detection, LOD), these are substitutes for the corresponding LOD/2. In the box and jitter plots, the 25–75 percentiles are drawn using a box; minimum and maximum are shown at the end of the thin lines (whiskers), while the median is marked as a horizontal line in the boxfitting. A Spearman’s rank correlation was performed to examine relationships between different TEs in muscle samples, and significant correlations (*p* < 0.05) are illustrated in Fig. 5. All the statistical analyses were performed using PAST software 3.22 (Hammer et al., 2001).

## Results

TE concentrations in the 15 muscular tissues of the three tern species collected from the two islands are reported in Table 1.

**Table 1:** Trace elements concentrations (µg g^−1^ dry weight (d.w.) ± standard deviations (SD)) referred to the number of individuals (*n*) grouped by the sampling sites and tern species, or total tissue samples (*n* = 15) analysed. Minimum (Min) and maximum (Max) referred to the total samples analysed; limit of quantification expressed in µg g^−1^, *BLQ* below limit of quantification

Elements (LOQ)		Jana Island			Karan Island		Total samples
		*T. bergii (n* = *4)*	*S. repressa (n* = *3)*	*O. anaethetus (n* = *3)*	*T. bergii (n* = *3)*	*O. anaethetus (n* = 2)*	*(n* = *15)*
Al (1.25)	Mean ± SD	76.31 ± 34.41	76.25 ± 50.63	29.75 ± 51.53	17.83 ± 11.84	BLQ–30.00	47.12 ± 43.27
	Median (Min–Max)	72.00 (44.75–116.50)	97.25 (18.50–113.00)	BLQ (BLQ–89.25)	15.25 (7.50–30.75)		30.75 (BLQ–116.50)
As (2.50)	Mean ± SD	3.22 ± 4.35	3.77 ± 3.69	3.77 ± 3.96	2.72 ± 4.71	BLQ–4.75	3.23 ± 3.54
	Median (Min–Max)	1.84 (BLQ–9.21)	3.95 (BLQ–7.37)	3.42 (BLQ––7.89)	BLQ (BLQ–8.16)		3.42 (BLQ–9.21)
Ba (0.04)	Mean ± SD	10.19 ± 15.68	3.97 ± 1.39	2.19 ± 1.02	1.77 ± 0.25	0.76–6.33	4.78 ± 8.14
	Median (Min–Max)	2.78 (1.52–33.67)	3.29 (3.04–5.57)	2.03 (1.27–3.29)	1.77 (1.52–2.03)		2.03 (0.76–33.67)
Ca (0.06)	Mean ± SD	93,037.50 ± 39,278.84	100,766.67 ± 29,507.27	78,733.33 ± 38,931.91	68,508.33 ± 25,869.97	33,200.00–114475.00	84,256.67 ± 34,039.2
	Median (Min–Max)	92,900.00 (61,275.00–129,425.00)	104,675.00 (69,500.00–128,125.00)	86,225.00 (36,600.00–113,375.00)	58,300.00 (49,300.00–97,925.00)		86,225.00 (33,200.00–129,425.00)
Cu (0.50)	Mean ± SD	5.67 ± 1.83	10.31 ± 4.4	4.98 ± 1.11	3.82 ± 1.56	3.73–5.87	5.97 ± 3.1
	Median (Min–Max)	5.07 (4.27–8.27)	9.07 (6.67–15.20)	5.33 (3.73–5.87)	3.20 (2.67–5.60)		5.60 (2.67–15.20)
Fe (0.13)	Mean ± SD	314.56 ± 75.49	343.42 ± 55.05	294.92 ± 36.46	218.58 ± 55.48	241.00–461.50	302.10 ± 79.26
	Median (Min–Max)	310.13 (260.50–396.75)	355.75 (283.25–391.25)	275.00 (275.00–337.00)	212.75 (166.25–276.75)		276.75 (166.25–396.75)
Hg (0.001)	Mean ± SD	0.127 ± 0.105	0.080 ± 0.035	0.046 ± 0.012	0.029 ± 0.01	0.068–0.125	0.078 ± 0.065
	Median (Min–Max)	0.090 (0.051–0.282)	0.084 (0.043–0.113)	0.043 (0.036–0.059)	0.025 (0.021–0.040)		0.059 (0.021–0.282)
K (1.25)	Mean ± SD	3757.50 ± 861.97	4181.67 ± 1028.79	4316.67 ± 895.91	4441.67 ± 482.33	3920.00–5395.00	4211.00 ± 798.27
	Median (Min–Max)	3417.50 (3185.00–5010.00)	4192.50 (3147.5–5205.00)	4270.00 (3445.00–5235.00)	4237.50 (4095.00–4992.50)		4192.50 (3147.50–5395.00)
Mg (0.05)	Mean ± SD	3226.88 ± 854.42	3099.17 ± 935.98	2847.50 ± 846.77	2377.50 ± 505.17	1300.00–3797.50	2865.17 ± 870.45
	Median (Min–Max)	3133.75 (2395.00–4245.00)	2865.00 (2302.50–4130.00)	2930.00 (1962.50–3650.00)	2392.50 (1865.00–2875.00)		2865.00 (1300.00–4245.00)
Mn (0.13)	Mean ± SD	6.97 ± 2.22	11.40 ± 1.61	5.35 ± 2.19	4.65 ± 0.92	2.11–7.89	6.81 ± 3.14
	Median (Min–Max)	6.05 (5.53–10.26)	11.05 (10.00–13.16)	6.05 (2.89–7.11)	4.74 (3.68–5.53)		6.05 (2.11–13.16)
Na (0.63)	Mean ± SD	8835.00 ± 2929.74	8843.33 ± 3155.32	6564.17 ± 1420.72	5610.83 ± 250.15	3432.50–7000.00	7255.17 ± 2557.85
	Median (Min–Max)	8900.00 (5895.00–11,645.00)	7217.50 (6832.5–12,480.00)	6205.00 (5357.50–8130.00)	5740.00 (5322.50–5770.00)		6755.00 (3432.50–12,480.00)
Pb (1.25)	Mean ± SD	BLQ	BLQ	1.87 ± 3.23	BLQ	BLQ	0.37 ± 1.45
	Median (Min–Max)			BLQ (BLQ–5.60)			BLQ (BLQ–5.60)
Sr (0.06)	Mean ± SD	492.10 ± 597.12	106.14 ± 11.96	140.83 ± 90.66	80.91 ± 33.53	37.50–546.14	235.71 ± 341.89
	Median (Min–Max)	247.16 (103.64–1370.45)	102.27 (96.59–119.55)	177.95 (37.50–207.05	81.59 (47.05–114.09)		114.09 (37.50–1370.45)
Zn (0.25)	Mean ± SD	135.06 ± 27.36	200.43 ± 34.67	116.88 ± 44.28	108.14 ± 21.75	41.82–164.94	134.89 ± 50.06
	Median (Min–Max)	138.57 (98.44–164.68)	188.57 (173.25–239.48)	111.43 (75.58–163.64)	96.10 (95.06–133.25)		137.40 (41.82–239.48)

*The two concentration values obtained are reported

**Fig. 2:**
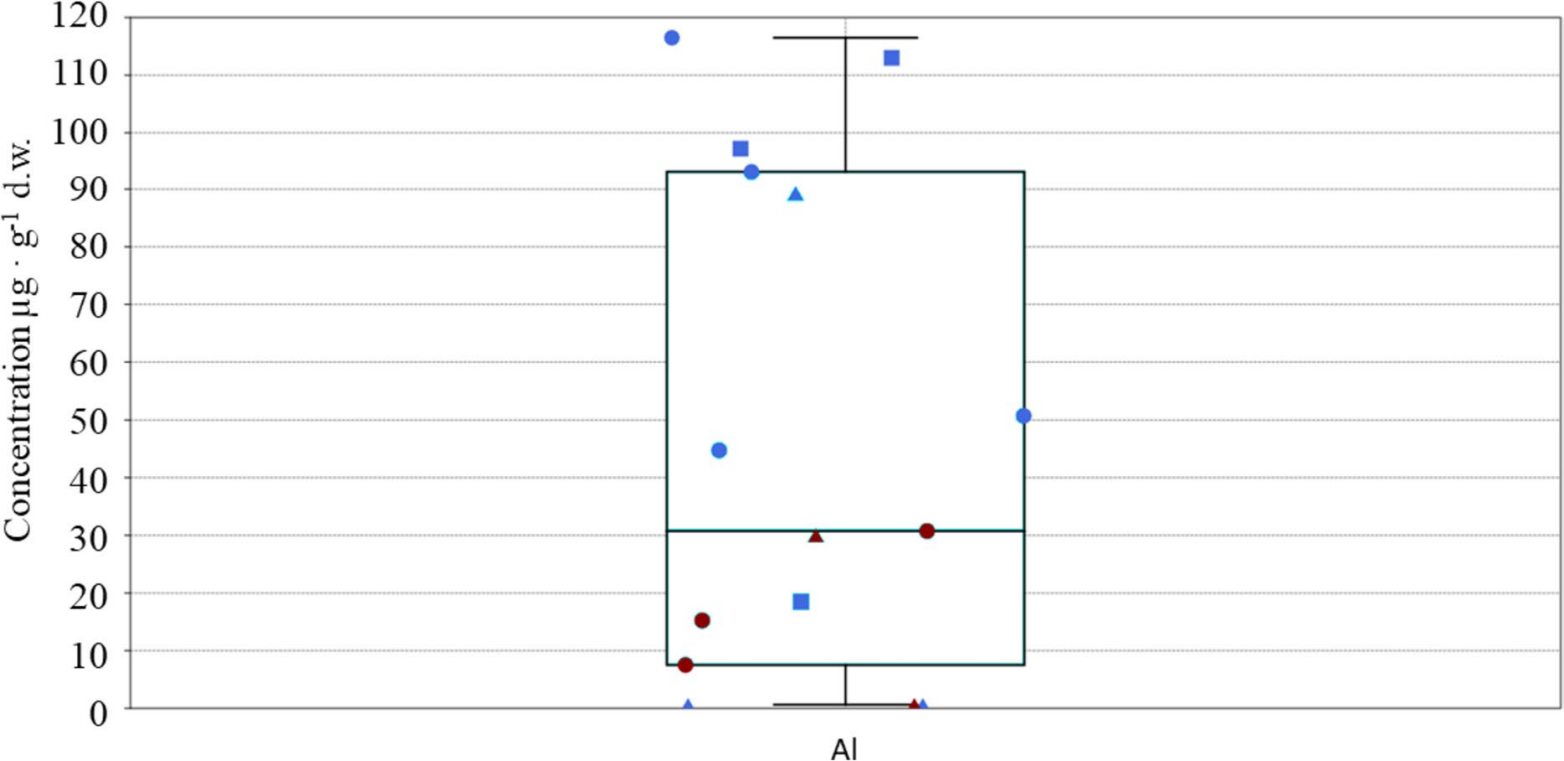
Box and jitter plot showing the concentrations of Al (minimum value corresponds to LOD/2 = 1.25 µg g^−1^) found in muscle tissues of *T. bergii* (dot), *S. repressa* (square), and *O. anaethetus* (triangle) collected from Jana Is. (blue symbols) and Karan Is. (red symbols)

Fifteen out of nineteen elements taken into consideration were detected in tern muscular tissues (Cd, Co, Cr, Ni and V which were below the limit of detection (LOD)). Al was not detected in three out of fifteen tissues. The analysis of Al showed higher values in *T. bergii* (mean = 76.31 µg g^−1^ d.w.; maximum = 116.5 µg g^−1^ d.w.) and *S. repressa* (mean = 76.25 µg g^−1^ d.w.; maximum: 113 µg g^−1^ d.w.) from Jana Is. compared to *O. anaethetus* from the same site (mean = 29.75 µg g^−1^ d.w.) with which Al was detected in only one out of three samples analysed (89.25 µg g^−1^ d.w.) (Table 1 and Fig. 2).

The concentrations of Al assessed in the specimens of the three tern species from Jana Is. were higher (mean = 62.33 µg g^−1^ d.w) than those found in the individuals from Karan Is. (mean = 16.70 µg g^−1^ d.w.) (Fig. 2 and Table 1).

**Fig. 3:**
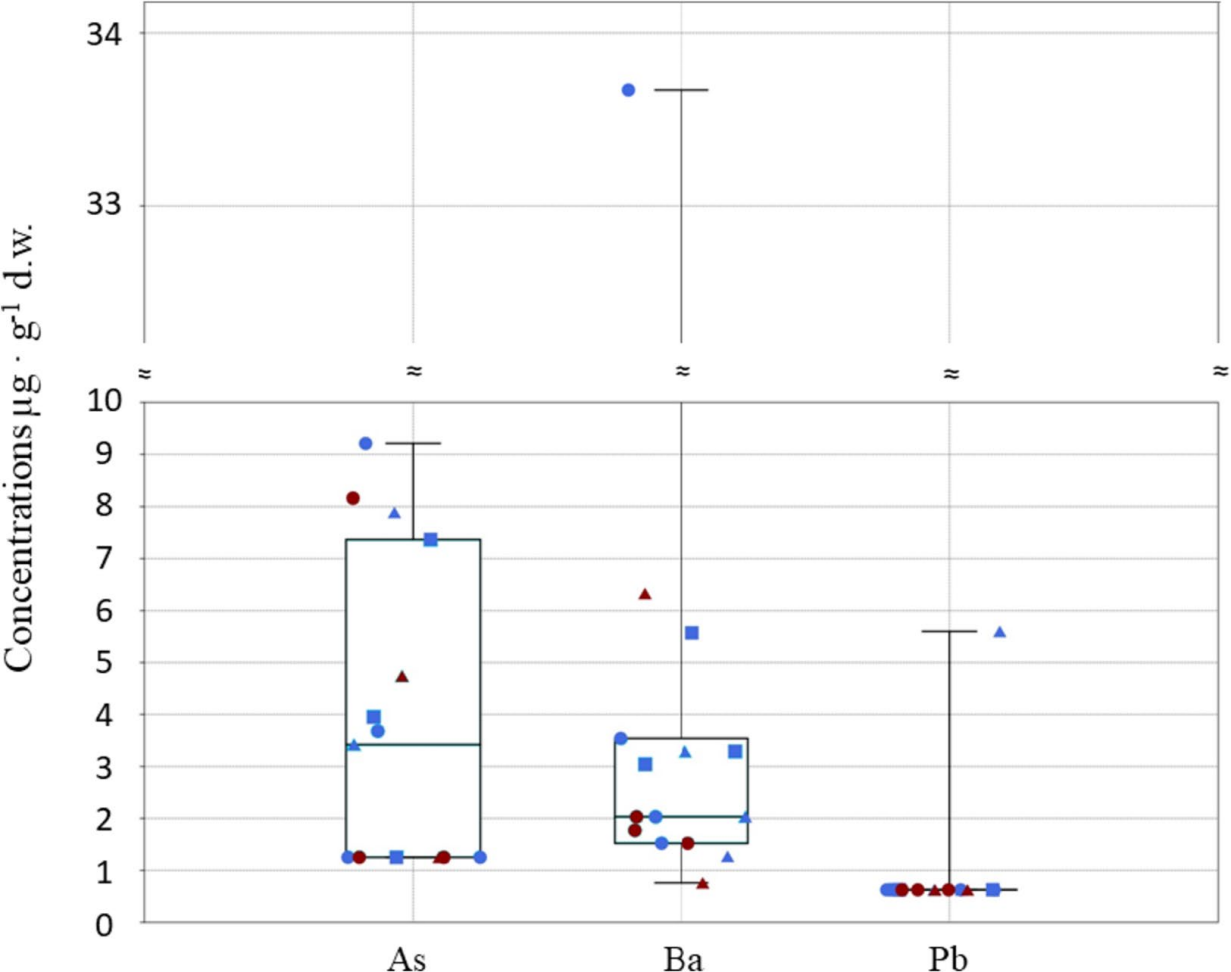
Box and jitter plot showing the concentrations of arsenic, barium and lead found in muscle tissues of *T. bergii* (dot)*, S. repressa* (square) and *O. anaethetus* (triangle) collected from Jana Is. (blue symbols) or Karan Is. (red symbols). For As and Pb, minimum concentration values correspond to LOD/2 (As = 1.25 µg g^−1^; Pb = 0.625 µg g^−1^)

Arsenic (As) was detected in seven out of fifteen tissues, with slightly more contaminated individuals from Jana Is. (mean = 3.55 µg g^−1^ d.w.; maximum = 9.21 µg g^−1^ d.w. in *T. bergii*), compared to those of the same species collected from Karan Is. (mean = 2.58 µg g^−1^ d.w.; maximum = 8.16 µg g^−1^ d.w.) (see Fig. 3 and Table 1).

While Ba was detected in all samples (mean = 4.78 µg g^−1^ d.w.; minimum = 0.76 µg g^−1^ d.w. in *O. anaethetus* from Karan Is.; maximum = 33.67 µg g^−1^ d.w. in *Thalasseus bergii* from Jana Is.) (see Fig. 3 and Table 1), Pb was detected in only one sample of *O. anaethetus* from Jana Is., with a concentration of 5.6 µg g^−1^ d.w. (see Fig. 3 and Table 1).

**Fig. 4:**
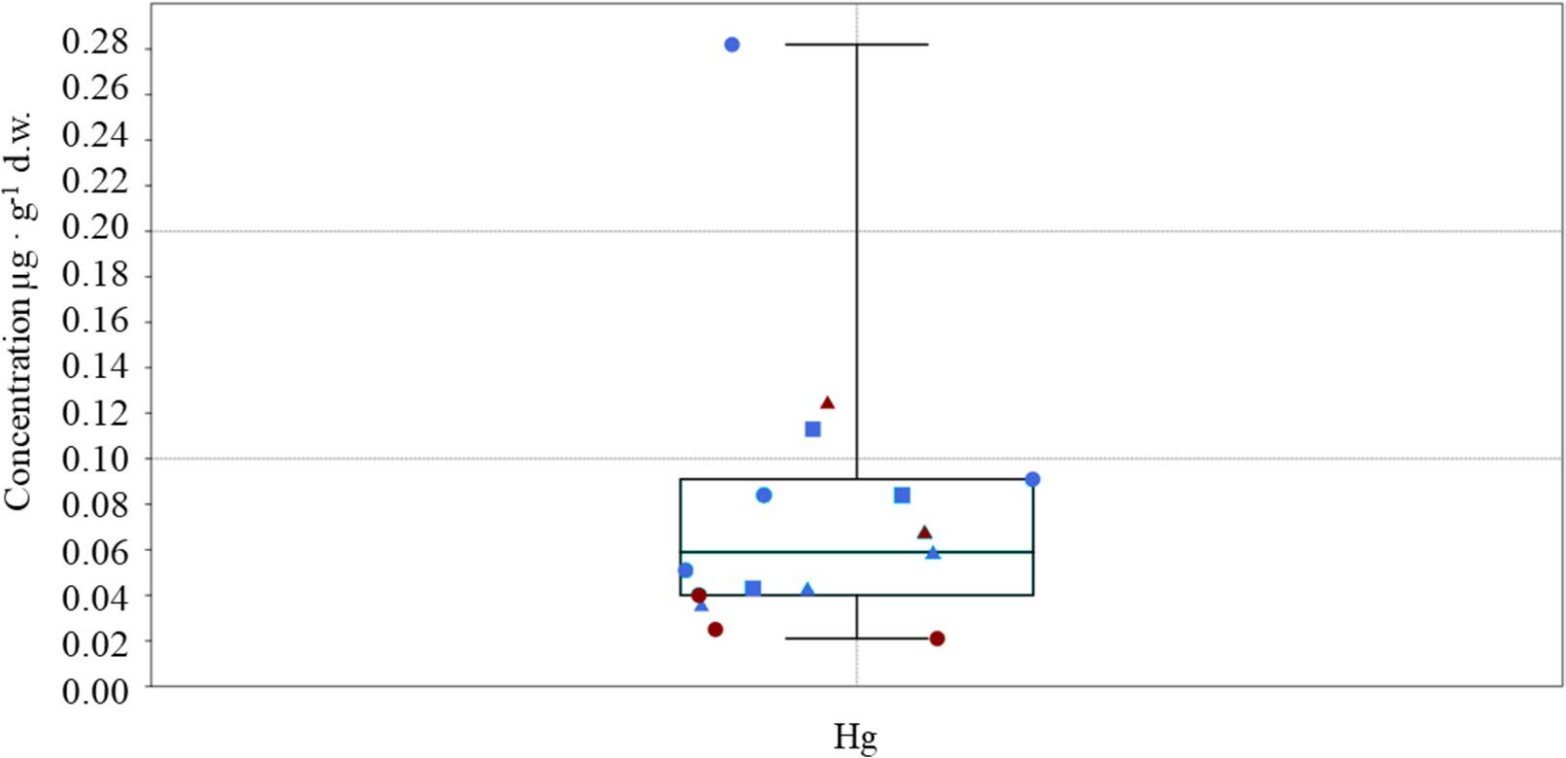
Box and jitter plot showing the concentrations of mercury found in muscle tissues of *T. bergii* (dot), *S. repressa* (square) and *O. anaethetus* (triangle) collected from Jana Is. (blue symbols) or Karan Is. (red symbols)

Differently, Hg was detected in all the analysed samples, with the highest average (0.127 µg g^−1^ d.w.) and maximum (0.282 µg g^−1^ d.w.) values found in *T. bergii* (see Fig. 4 and Table 1).

Likewise, the other TEs analysed (Cu, Fe, K, Mn, Mg, Na, Sr, Zn) were detected in all samples. In general, all individuals showed high variability in terms of contamination of different TEs (see high SD values compared with mean concentration in Table 1).

Nevertheless, considering the 14 TEs detected, no significant differences in contamination profiles were found between the two sites (PERMANOVA: *p* = 0.2107) and different species (PERMANOVA: *p* = 0.0978), and the interaction of the latter two factors (PERMANOVA: *p* = 0.2974).

**Fig. 5:**
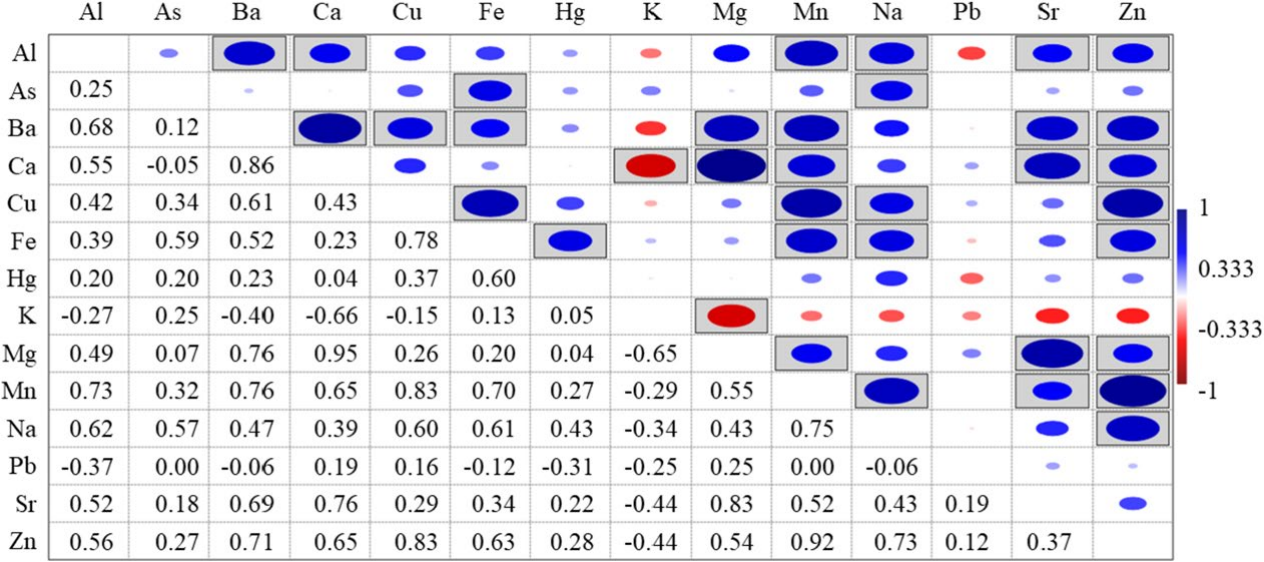
Plot of Spearman correlation test showing significant correlations (*p* < 0.05) indicated as disk marks in the plot and related statistical values

The Spearman’s rank analysis highlighted strong (rs = 0.6–0.79) or very strong (rs = 0.8–1.0) correlations (Akoglu, 2018) in 35 of the possible 91 combinations (Fig. 5).

The strongest positive correlations were between Ca and Ba (r_s_ = 0.86), Ca and Mg (r_s_ = 0.95), Cu and Mn (r_s_ = 0.83), Sr and Mg (r_s_ = 0.83), Zn and Cu (r_s_ = 0.83) and Zn and Mn (r_s_ = 0.92).

## Discussion

The toxicity of TEs can have an impact on the health of adult seabirds and potentially on their progeny or breeding success as observed by Lam et al. (2005). Information on the concentration levels of these elements in terns is fragmentary. Only a few research studies focused on the tissues of *Laridae* as reported for lead contamination in the review of Korbecki et al. (2019) or Hg by Zamani-Ahmadmahmoodi et al. (2014), and no studies have been conducted on tissues of *T. bergii*, *S. repressa* and *O. anaethetus*.

However, several studies have investigated the trace elements in the muscle tissues of various seabird species of which Table 2 reported the concentration levels of different elements.

**Table 2:** Concentration levels of trace elements in µg g^−1^ d.w. in muscles of waterbirds. The levels of K, Mg, Na and Ca in this paper have not been added to the table as they have not been invested in the other studies reported

Family	Scientific name	No	Fe	Sr	Zn	Cu	Al	Pb	Mn
Laridae	*Sterna bergii*	7	273.43	315.88	123.53	4.88	51.25	0.63	5.98
	*Sterna repressa*	3	343.42	106.14	200.43	10.31	76.25	0.63	11.40
	*Onychoprion anaethetus*	5	317.45	201.23	111.48	4.91	24.23	1.62	5.21
	*Larus michahellis*	6	17450	NA	5113.4	1642.40	2558.4	179.6	168.4
	*Larus crassirostris*	5	NA	0.19	71.70	14.8	NA	0.011	1.83
Rallidae	*Fulica atra*	6	22182.60	NA	3135.6	273.85	2711.60	125.6	127
	*Gallinula chloropus*	6	NA	8.27	NA	NA	NA	0.8	NA
Procellariidae	*Pterodroma baraui*	20	404.00	NA	101.00	27.7	NA	NA	1.65
	*Puffinus lherminieri*	23	365.00	NA	73	21	NA	NA	1.83
Phaethontidae	*Phaethon lepturus*	32	367.00	NA	86.70	28.1	NA	NA	2.33
Phalacrocoracidae	*Phalacrocorax carbo*	6	NA	5.30	NA	NA	NA	0.30	NA
Podicipedidae	*Podiceps cristatus*	10	NA	7.06	NA	NA	NA	0.72	NA
Anatidae	*Anas clypeata*	8	NA	6.35	NA	NA	NA	0.53	NA
	*Anas crecca*	20	31.82*	NA	8.72*	NA	NA	0.27*	NA
	*Mareca strepera*	20	65.32*	NA	8.14*	NA	NA	0.95*	NA
	*Anser anser*	9	NA	NA	93.8	43	11.6	NA	NA
Recurvirostridae	*Himantopus himantopus*	10	NA	6.96	NA	NA	NA	0.58	NA
Scolopacidae	*Tringa stagnatilis*	6	NA	7.2	NA	NA	NA	0.63	NA
	*Calidris canutus*	3	NA	NA	39.6	31.1	2.5	NA	NA
Charadriidae	*Vanellus vanellus*	2	NA	7.2	NA	NA	NA	0.60	NA
Threskiornithidae	*Platalea leucorodia*	2	NA	7.10	NA	NA	NA	0.70	NA
Ardeidae	*Ardeola grayii*	3	NA	NA	0.39	0.05	NA	4.85	NA
	*Nycticorax nycticorax*	3	NA	NA	1.26	0.54	NA	5.39	NA
Charadriidae	*Pluvialis squatarola*	8	NA	NA	92.8	51	17.2	NA	NA

Family	As	Hg	Ba	Cd	Co	Cr	Ni	V	Location	Ref
Laridae	3.72	0.08	6.58	< 0.1	< 0.3	< 0.3	< 0.6	< 0.6	Saudi Arabia	1
	4.19	0.08	3.97	< 0.1	< 0.3	< 0.3	< 0.6	< 0.6	Saudi Arabia	1
	3.71	0.07	2.73	< 0.1	< 0.3	< 0.3	< 0.6	< 0.6	Saudi Arabia	1
	98.00	59.6	0.95	< 0.001	5.00	254.8	366	3.40	Turkey	2
	NA	0.40	0.11	0.654	0.07	2.9	NA	0.042	Japan	3
Rallidae	49.8	30.40	32.2	0.1	7.4	93.68	187.4	2.4	Turkey	2
	NA	NA	NA	0.67	NA	NA	4.03	NA	Iran	4
Procellariidae	NA	2.84	NA	9.28	NA	NA	NA	NA	Réunion, Indian Ocean	5
	NA	1.16	NA	4.55	NA	NA	NA	NA	Réunion, Indian Ocean	5
Phaethontidae	NA	0.75	NA	3.67	NA	NA	NA	NA	Réunion, Indian Ocean	5
Phalacrocoracidae	NA	NA	NA	0.40	NA	NA	2.57	NA	Iran	4
Podicipedidae	NA	NA	NA	0.54	NA	NA	3.42	NA	Iran	4
Anatidae	NA	NA	NA	0.48	NA	NA	3.00	NA	Iran	4
	NA	NA	NA	0.1*	NA	0.08*	NA	NA	Iran	6
	NA	NA	NA	0.35*	NA	0.16*	NA	NA	Iran	6
	0.22	0.08	NA	NA	NA	NA	1.8	NA	France	7
Recurvirostridae	NA	NA	NA	0.52	NA	NA	3.3	NA	Iran	4
Scolopacidae	NA	NA	NA	0.57	NA	NA	3.53	NA	Iran	4
	3.1	0.68	NA	NA	NA	NA	0.3	NA	France	7
Charadriidae	NA	NA	NA	0.60	NA	NA	3.5	NA	Iran	4
Threskiornithidae	NA	NA	NA	0.60	NA	NA	3.20	NA	Iran	4
Ardeidae	0.1	0.3	NA	NA	NA	1.12	0.56	NA	South India	8
	1.92	0.01	NA	NA	NA	0.72	0.54	NA	South India	8
Charadriidae	7.7	0.48	NA	NA	NA	NA	0.7	NA	France	7

*Median values; the values preceded by the symbol < refer to the limits of quantification; TEs in seabird muscles found by Durmaz et al. were converted to dry weight from wet weight considering 75% water (Listrat et al., 2016). *NA* not available. References list: 1, this study; 2, Durmaz et al., 2017; 3, Agusa et al., 2005; 4, Dahmardeh Behrooz & Burger, 2022; 5, Kojadinovic et al., 2007; 6, Sinkakarimi et al., 2018; 7, Lucia et al., 2010; 8, Pandiyan et al., 2022

Several studies investigate these pollutants in eggs or feathers of seabirds (Khademi et al., 2015; Kitowski et al., 2018; Lam et al., 2005; Thongcharoen et al., 2018; Zolfaghari et al., 2007). For example, a study conducted in the Gulf showed high contamination of TEs (As, Cd, Co, Pb, Ni, Se and V) in eggs of *O. anaethetus*, and *S. bengalensis* (Khademi et al., 2015). In particular, all the elements in common with our research showed higher levels of concentration in both the shells of the eggs and their contents. The reason for this higher contamination could be due to geographically different sampling sites. The islands of Jana and Karan are located about 46 and 80 km respectively offshore from the Jubail fishing port, near Jubail Marine Wildlife Sanctuary, and ~ 40 km from each other (Maneja et al., 2021); the egg study was carried out in Khure Musa estuary, northwest of the Gulf. The latter site is highly exposed to metals and semimetals since there are big petrochemical plants including oil and gas mining, agricultural land and various industrial activities (Khademi et al., 2015).

The elements analysed in our study showed that the highest levels are in descending order as follows: Ca > Na > K > Mg > Fe > Sr > Zn > Al > Mn > Cu > Ba > As > Pb > Hg. This trend is partially different to that observed in Turkey in two waterbirds (the Eurasian coot and the yellow-legged gull) by Durmaz et al. (2017) where levels of several orders of magnitude higher were found and in which the order of average concentrations of the common elements was as follows: Fe > Zn > Cu > Al > Pb > Mn > As > Hg > Ba. While our study showed that Cd, Co, Cr, Ni and V were below the permissible limits, Durmaz et al. (2017) found high concentrations of these same elements, especially for Ni. The undetectability of these elements in the Jana and Karan Is. suggests less contamination of these sites and therefore less environmental concerns.

In our work, Ba levels were higher than those found in other muscle tissues of *Laridae* by Agusa et al., (2005)*.* A previous study conducted in the same region showed high concentrations of Ba in the sediments of vegetated habitats including seagrass and mangroves (Rabaoui et al., 2020). Considering that birds can be exposed to Ba through water sources (such as food or water) or sediment, attention should be paid to the levels of Ba present in the environment.

The recorded levels for essential elements such as Zn and Mn were found to be similar to those found in the muscles of other *Laridae* by Agusa et al., (2005) and in the muscles of three pelagic seabirds species by Kojadinovic et al. (2007). In the latter work, Fe and Cu levels resulted similar to those found in our study*.* Conversely, high levels of Ca and Sr are probably explained both by metabolic needs and by the high levels of concentration found at nesting sites observed for Karan Is. by Basyoni (1999). Both elements are fundamental during egg production resulting in increased Ca absorption as well as increased absorption of Sr (Zhang & Ma, 2011). Like Ca, other essential macrominerals such as Na, Mg and K showed high concentration levels. Such integral elements are necessary within the body to maintain the ionic balance of structural compounds, amino acids and nucleic acids (Briffa et al., 2020).

At high levels, essential elements may also have adverse effects on reproduction or toxic effects such as on the kidneys as observed by Lucia et al. (2010) for Cu and Zn; therefore, the continuous monitoring of these elements, generally not analysed or reported in the biomonitoring works, is fundamental to understanding the state of health of the organisms. In the same way, the analysis of toxic elements, dangerous even at low concentrations such as, Hg, Pb, Al, Cd and Cr, is equally necessary for the monitoring of ecosystems.

Arsenic showed levels of concentration of the same order of magnitude as in the muscles of other *Laridae* and *Sternidae* (Savinov et al., 2003) or other seabirds (Lucia et al., 2010).

The levels of Hg found in our study (mean of 15 samples = 0.078 µg g^−1^ d.w.) are lower than those found in different tissues, including muscles (greater than 1 µg g^−1^ d.w.) of two seabird species (Common Tern and Slender-billed Gull) from the north-western corner of the Gulf (Zamani-Ahmadmahmoodi et al., 2014).

In the present study, Pb was detected only in one sample of *O. anaethetus* of Jana Is. at a concentration of 5.6 µg g^−1^ d.w. Although these concentration values do not exceed the threshold level for toxic effects (Pb > 6.0 µg g^−1^ d.w. in the liver, Kim & Oh, 2017), this value is higher than that found on average in different *Laridae* and *Sternidae* in Asia (Agusa et al., 2005; Hoshyari et al., 2012; Kim & Oh, 2017) and North America (Borgå et al., 2006; Maedgen et al., 1982) studies.

In our work, levels found for Al were higher than those found in other waterbirds (Kim & Oh, 2017; Kim et al., 2013; Lucia et al., 2010). High levels of Al can cause negative effects on the reproductive system, calcium homeostasis, and phosphorus metabolism that lead to muscle weakness and decreased growth rates (Lucia et al., 2010).

Human activities contribute to Al levels (from coal combustion, mining, waste incineration and motor vehicle exhaust) in air and water. In addition, since Al is highly soluble in an acidic environment, acid rain can cause the amount of Al dissolved in the surrounding water to increase (Alasfar & Isaifan, 2021). An important input source of Al comes from the sediments of coastal areas, as observed in areas adjacent to Karan and Jana Is. (Al-Jubail area, Al_sediment_ mean = 1887.07 µg g^−1^; range = 555–3602 µg g^−1^ (El-Sorogy et al., 2018)) or on the coast of Al-Khobar located in the south of the Saudi coast in the Gulf (Al mean = 2041 µg g^−1^; range = 688–3224 µg g^−1^; Alharbi et al., 2017)) suggesting that the levels we found in our study may be due to bioaccumulation phenomena or accidental ingestion of contaminated material.

The analysis of these elements, particularly the non-essential ones (e.g. Al, As, Cd, Hg and Pb), also provides implicit indications of the possible levels of contamination of the trophic network to which these species belong. Usually, due to bioaccumulation and biomagnification effects, the levels recorded between organisms belonging to the same food chain are correlated (Durmaz et al., 2017). In this context, seabirds are excellent bioindicators of exposure to TEs, and they can provide useful information comparable with other similarly exposed organisms evaluated according to threshold limits exposure criteria (Durmaz et al., 2017).

The PERMANOVA analysis showed that there are no significant differences in the contamination profiles observed in the different species of the two sites. This result suggests that the levels of exposure to TEs for the different individuals in this study, except for a few cases, are similar, probably due to the geographical proximity of the two islands.

The stronger correlations shown by Spearman’s analysis highlight potential physiological meanings. Ca, Mg and Sr are closely chemically related and have all been implicated in both health and musculoskeletal and cardiovascular diseases (Curtis et al., 2021). The correlation found between Ca and Mg may arise from the role that Mg has in regulation (e.g. muscle contraction, e.g. Mg stimulates Ca resorption) or modulation of Ca transport at the cellular level (Jahnen-Dechent & Ketteler, 2012). The positive correlation between Ba and Ca suggests that the two vicariant elements are in regulated equilibrium. The strong positive correlations between Cu and Mn, Cu and Zn and Zn and Mn suggest that these essential elements are in homeostatic proportionality ratio. The balancing of these elements, of fundamental intake in the diet, is essential in the tissues, for example, Cu and Zn which are also structural ions of different enzymes. Zinc deficiency and Cu excess have been reported to be associated with inflammation processes (Abolbashari et al., 2018).

Overall, the results showed that the levels found were in line with other works; however, it was not possible to make a comparison with tissues of the same species we analysed. Considering the interspecific variability of bioaccumulation of these pollutants, this research work is a first point of reference for subsequent and similar research. Despite the low number of samples analysed, the high concentration variability found for some TEs suggests that each individual may be subject to several contamination episodes (e.g. sporadic ingestion of contaminated food).

In this regard, our results should be further confirmed by similar biomonitoring studies, investigating the main sources of contamination on environmental matrices in nesting and foraging sites (such as water, soil and sediment) and on the main prey on which the terns feed on. This requires regular monitoring to assess whether these levels are increasing or decreasing in order to prevent potential risks.

## Conclusions

Pollutants affect the environmental quality and concomitant development of diseases and imbalances of physiological parameters. This work provides the first concentration data of different elements analysed in muscle tissues of three species of seabirds from two Saudi islands in the Gulf. The analyses revealed for several elements that the levels recorded are of the same order of magnitude as those found in other works, suggesting that the levels of bioaccumulation are similar. For some of these elements, despite the high concentrations recorded, the results do not give rise to particular concern because they are essential elements that can be regulated by organisms. The remoteness from the sources of contamination would justify the relatively low state of contamination supporting the good conservation status of ecosystems. However, particular attention is paid to non-essential elements, such as Al, Ba or Pb, which, in some cases, showed levels higher than those usually recorded in other works. In this context, gaps persist in our understanding of the specific TEs affecting tern populations and their nesting and feeding habitats. These preliminary results indicate TEs levels in these species; however, further surveys and sampling should be conducted to support and evaluate these contamination trends over time. The study of these indicators of TEs pollution in aquatic ecosystems deserves increasing attention because they allow us to assess the current state of pollution and support actions aimed at eliminating or at least reducing the sources of pollution.

### Supplementary information

Below is the link to the electronic supplementary material.

Supplementary file1 (DOCX 16 KB)

## Data Availability

Data will be made available upon request.
