# Supplementary material for: Trace element levels in the muscles of three tern species (Aves: Laridae) from the western Arabian Gulf: environmental assessment and implications for conservation
Source: Environ Monit Assess. 2024 Feb 5;196(3):235. doi: 10.1007/s10661-024-12385-9 (PMC10844429; doi:10.1007/s10661-024-12385-9)
Supplement: Supplementary file 1 — Supplementary file1 (DOCX 16 KB) [file 10661_2024_12385_MOESM1_ESM.docx]

**Trace element levels in the muscles of three tern species (Aves: Laridae) from the western Arabian/Persian Gulf: environmental assessment and implications for conservation**

Lamia Yacoubi ^a^, Dario Savoca ^b,c,*^, Radhouan Belgacem El Zrelli ^d^, Jinoy Gopalan ^e^, Mazen Nazal ^e^, Yu-Jia Lin ^f^, Antonella Maccotta ^b,c^, Foued Hamza ^g^, Md. Simul Bhuyan ^h^, Marco Arculeo ^b^, Lotfi Jilani Rabaoui ^a,g^

^a^ *University of Tunis El Manar, Faculty of Science of Tunis, Laboratory of Biodiversity & Parasitology of Aquatic Ecosystems (LR18ES05), University Campus, 2092 Tunis, Tunisia*

^b^ *Dipartimento di Scienze e Tecnologie Biologiche, Chimiche e Farmaceutiche (STEBICEF), Università degli Studi di Palermo, Via Archirafi 20, Palermo 90133, Italy*

^c^ *NBFC, National Biodiversity Future Center, Palermo, 90123, Italy*

^d^ *SADEF Agronomy & Environment, 30 Rue de la Station, 68700 Aspach-Le-Bas, France*

^e^ *Applied Research Center for Environment and Marine Studies, Research Institute, King Fahd University of Petroleum and Minerals (KFUPM), Dhahran 31261, Saudi Arabia*

^f^ *National Taiwan University, Institute of Oceanography, Taipei 10617, Taiwan*

^g^ *National Center for Wildlife, Ministry of Environment, Water & Agriculture, Riyadh, Saudi Arabia*

^h^ *Bangladesh Oceanographic Research Institute, Cox’s Bazar-4730, Bangladesh*

*** Corresponding Author:** Dario Savoca (Email: dario.savoca@unipa.it)

Table S1. Analysis of Dorm-2 certified standard materials (certified and measured values (µg · g^-1^), and recovery (%)).

| **Elements** | **Certified** | **Measured** | **Recovery** |
| --- | --- | --- | --- |
| Al | 10.9±1.7 | 11.45 | 105 |
| As | 18.0±1.1 | 24.84 | 138 |
| Ba | - | - | - |
| Ca | - | - | - |
| Cd | 0.043±0.008 | 0.040 | 92 |
| Co | 0.182±0.031 | 0.206 | 113 |
| Cr | 34.7±5.5 | 37.13 | 107 |
| Cu | 2.34±0.16 | 2.06 | 88 |
| Fe | 142±10 | 134.9 | 95 |
| Hg | 4.64±0.26 | 3.85 | 83 |
| K | - | - | - |
| Mg | - | - | - |
| Mn | 3.66±0.34 | 4.32 | 116 |
| Na | - | - | - |
| Ni | 19.4±3.1 | 20.37 | 105 |
| Pb | 0.065±0.007 | 0.059 | 91 |
| Sr | - | - | - |
| V | - | - | - |
| Zn | 25.6±2.3 | 24.58 | 96 |
